# Partnerships and Community Engagement Key to Policy, Systems, and Environmental Achievements for Healthy Eating and Active Living: a Systematic Mapping Review

**DOI:** 10.5888/pcd19.210466

**Published:** 2022-08-25

**Authors:** Leslie Cunningham-Sabo, Angela Tagtow, Sirui Mi, Jessa Engelken, Kiaya Johnston, Dena R Herman

**Affiliations:** 1 Colorado State University, Food Science and Human Nutrition, Fort Collins, Colorado; 2 Colorado School of Public Health, Community and Behavioral Health, Aurora, Colorado; 3 Äkta Strategies, LLC, Elkhart, Iowa; 4 University of Washington, School of Public Health, Nutritional Sciences Program, Seattle, Washington; 5 University of California Los Angeles, Fielding School of Public Health, Los Angeles, California; 6 California State University Northridge, Nutrition, Dietetics, and Food Science, Northridge, California

## Abstract

**Introduction:**

Policy, systems, and environmental (PSE) change approaches frequently address healthy eating and active living (HEAL) priorities. However, the health effects of PSE HEAL initiatives are not well known because of their design complexity and short duration. Planning and evaluation frameworks can guide PSE activities to generate collective impact. We applied a systematic mapping review to the Individual plus PSE Conceptual Framework for Action (I+PSE) to describe characteristics, achievements, challenges, and evaluation strategies of PSE HEAL initiatives.

**Methods:**

We identified peer-reviewed articles published from January 2009 through January 2021 by using CINAHL, Web of Science, MEDLINE, PsycINFO, and CAB Abstracts databases. Articles describing implementation and results of PSE HEAL initiatives were included. Activities were mapped against I+PSE components to identify gaps in evaluation efforts.

**Results:**

Independent reviewers examined 437 titles and abstracts; 52 peer-reviewed articles met all inclusion criteria. Twenty-four focused on healthy eating, 5 on active living, and 23 on HEAL. Descriptive analyses identified federal funding of initiatives (typically 1–3 years), multisector settings, and mixed-methods evaluation strategies as dominant characteristics. Only 11 articles reported on initiatives that used a formal planning and evaluation framework. Achievements focused on partnership development, individual behavior, environmental or policy changes, and provision of technical assistance. Challenges included lack of local coalition and community engagement in initiatives and evaluation activities and insufficient time and resources to accomplish objectives. The review team noted vague or absent descriptions of evaluation activities, resulting in questionable characterizations of processes and outcomes. Although formation of partnerships was the most commonly reported accomplishment, I+PSE mapping revealed a lack of engagement assessment and its contributions toward initiative impact.

**Conclusion:**

PSE HEAL initiatives reported successes in multiple areas but also challenges related to partnership engagement and community buy-in. These 2 areas are essential for the success of PSE HEAL initiatives and need to be adequately evaluated so improvements can be made.

**Summary**

**What is already known on this topic?**

Policy, systems, and environmental (PSE) change approaches are frequently used for healthy eating and active living (HEAL) initiatives. Results are often difficult to determine, however, because of their multicomponent designs and insufficient evaluation.

**What is added by this report?**

We used the Individual + PSE Conceptual Framework for Action to map HEAL initiatives and related evaluation efforts. Evaluation gaps were prevalent for assessing the strength of community and partnership engagement.

**What are the implications for public health practice?**

Frameworks that plan for and evaluate community engagement and partnerships, such as I+PSE, can support achievement of initiative goals.

**MEDSCAPE CME**

In support of improving patient care, this activity has been planned and implemented by Medscape, LLC and *Preventing Chronic Disease*. Medscape, LLC is jointly accredited by the Accreditation Council for Continuing Medical Education (ACCME), the Accreditation Council for Pharmacy Education (ACPE), and the American Nurses Credentialing Center (ANCC), to provide continuing education for the healthcare team.

Medscape, LLC designates this Journal-based CME activity for a maximum of 1.00 AMA PRA Category 1 Credit(s)™. Physicians should claim only the credit commensurate with the extent of their participation in the activity.

Successful completion of this CME activity, which includes participation in the evaluation component, enables the participant to earn up to 1.0 MOC points in the American Board of Internal Medicine’s (ABIM) Maintenance of Certification (MOC) program. Participants will earn MOC points equivalent to the amount of CME credits claimed for the activity. It is the CME activity provider’s responsibility to submit participant completion information to ACCME for the purpose of granting ABIM MOC credit.

Release date: August 25, 2022; Expiration date: August 25, 2023

**Learning Objectives**

Upon completion of this activity, participants will be able to:

1. Distinguish characteristics of research into healthy living
2. Assess actions in healthy living research
3. Analyze the most successful components of healthy living research
4. Evaluate lessons learned from healthy living research

**EDITOR**

Rosemarie Perrin

Editor

Preventing Chronic Disease 

Atlanta, GA

**CME AUTHOR**

Charles P. Vega, MD

Health Sciences Clinical Professor of Family Medicine

University of California, Irvine School of Medicine

Irvine, California

Charles P. Vega, MD, has the following relevant financial relationships:

Advisor or consultant for: GlaxoSmithKline; Johnson & JohnsonAUTHORS

**AUTHORS**

Leslie Cunningham-Sabo, PhD, RDN

Colorado State University, Food Science and Human Nutrition; Colorado School of Public Health, Community and Behavioral Health, Fort Collins, CO

Angie Tagtow, MS, RD, LD

Äkta Strategies, LLC, Elkhart, IA

Sirui Mi, MS, RDN

Colorado State University, Food Science and Human Nutrition, Fort Collins, Colorado

Jessa Engelken, MPH, RDN

University of Washington Seattle Campus, Public Health, Seattle, Washington

Kiaya Johnston

Colorado State University, Food Science and Human, Nutrition, Fort Collins, Colorado

Dena Herman, PhD, MPH, RD

University of California Los Angeles, Fielding School of Public Health; California State University Northridge, Nutrition, Los Angeles, California, and Northridge, California

## Introduction

Obesity prevention and other public health initiatives emphasize policy, systems, and environmental (PSE) change in addition to traditional approaches that focus on individuals (1,2). Federal PSE initiatives include the Centers for Disease Control and Prevention’s (CDC’s) State Physical Activity and Nutrition Program, High Obesity Program, and Racial and Ethnic Approaches to Community Health program and the US Department of Agriculture’s (USDA’s) Supplemental Nutrition Assistance Program Education (SNAP-Ed) and Expanded Food and Nutrition Education Program (EFNEP).

Evidence is lacking for the health impact of PSE initiatives because of their complex nature, insufficient capacity and resources for program implementation (3,4), and absence of robust evaluation strategies (5). Several theory-based models and frameworks have informed PSE initiatives, including the RE-AIM (Reach, Efficacy, Adoption, Implementation and Maintenance) model (6), a systems thinking framework (7), a collective impact framework (8), a policy adoption model (2), and SNAP-Ed (9), along with an emphasis on health equity (10,11). A new framework is the Individual Plus Policy, Systems, and Environmental Conceptual Framework for Action (I+PSE) (12), informed by CDC’s Social–Ecological Model (13) and the Contra Costa Health Services Spectrum of Prevention (14). I+PSE is unique in that it views determinants of health through social, commercial, and political lenses. It guides users to examine a range of tactics to produce sustainable and synergistic effects through 7 action components (Table 1).

**Table 1:** Components of the I+PSE Conceptual Framework for Action^a^

I+PSE action component	Definition for healthy eating and active living (HEAL)
1. Strengthen individual knowledge and skills	Enhance individual’s, or household’s decision-making and capability of participating in or benefitting from HEAL.
2. Promote community engagement and education	Connect with diverse groups of people to inform them about the benefits of HEAL and to establish bi-directional communication, trust, and support to advance HEAL approaches.
3. Activate intermediaries and service providers	Inform and educate intermediaries and service providers who transmit information about HEAL to others.
4. Facilitate partnerships and multisector collaborations	Foster relationships and cultivate multisector collaborations with stakeholders about individual, community, and/or population approaches to HEAL.
5. Align organizational policies and practices	Revise or adapt policies, procedures, and practices within institutions that support HEAL.
6. Foster physical, natural, and social settings	Design, foster, and maintain physical (built), natural (ecosystems), and social settings within institutions and public environments that support HEAL.
7. Advance public policy and legislation	Develop strategies to inform change to laws, regulations, and public policies (local, state, federal) that support HEAL.

Abbreviations: HEAL, healthy eating active living; I+PSE, Individual Plus Policy, Systems, and Environmental Framework for Action.

a Adapted from Tagtow et al (12).

1. Strengthen individual knowledge and skills
2. Promote community engagement and education
3. Activate intermediaries and service providers
4. Facilitate partnerships and multisector collaborations
5. Align organizational policies and practices
6. Foster physical, natural, and social settings
7. Advance public policy and legislation

I+PSE then addresses the necessity of complex evaluation strategies at multiple levels to identify outcomes and effects intended by these coordinated action components. The cyclical processes of assessment, planning, implementation, and evaluation are supported by systems thinking and reflection.

Our review characterizes activities implemented and evaluated in PSE HEAL (healthy eating and active living) initiatives by using I+PSE’s 7 action components to answer 5 questions:

1. What are the key characteristics of PSE HEAL initiatives?
2. How are the 7 I+PSE components represented in these initiatives?
3. How are achievements and challenges described?
4. How are initiative activities evaluated?
5. Are there gaps in evaluation, and if so, where?

## Methods

### Data sources

We chose a systematic mapping review approach because of its relevance for addressing our research questions. Mapping reviews categorize and map existing literature and are based on questions rather than topics (15,16). This type of review is the most appropriate design for assessing an abundance of diverse research. Such reviews can identify gaps in the area of interest and can act as a first step toward a traditional systematic review (17).

Criteria for articles included in our review were that they were published in English from January 2009 through January 2021, reported the implementation and evaluation of a HEAL initiative, and discussed PSE activities. Conference abstracts, reviews, and commentaries were excluded. We used Mendeley Reference Manager software, version 1.19.8 (Mendeley Ltd) for storage and sorting of retrieved documents and followed the criteria outlined in the Preferred Reporting Items for Systematic Reviews and Meta-Analyses (PRISMA) statement (18).

After consultation with a research librarian, authors completed the literature search by using these terms: “policy, systems, and environmental” OR “policy, systems, and environment” OR “policy, systems, environment” OR “policy, systems, environmental” OR “policy and environmental” AND evaluat* OR assess* OR initiative OR intervention OR framework. We searched CINAHL, Web of Science, MEDLINE, PsycINFO, and CAB Abstracts databases. An initial search was conducted in January 2020, and results were updated with a search in January 2021 following the same procedures.

### Study selection

After removing duplicate records, we reviewed search results in 3 phases. In the first phase, 1 researcher reviewed each record’s title and abstract. Records passed this phase if they contained any mention of PSE and dealt with a topic related to public health. All remaining documents were sorted into either “pass” or “fail” electronic folders.

In the second phase, the same researcher completed a more detailed review of the title and abstract records that passed the first review phase. During this step, articles were divided into 7 folders: 1) those eligible for full text review because their primary focus was PSE and HEAL, 2) those that did not specifically deal with PSE and HEAL, 3) those that described a PSE HEAL initiative’s protocol or methods but no intervention results, 4) those that discussed a PSE evaluation framework that did not include application to a specific initiative, 5) conference abstracts, 6) reviews, and 7) commentaries. Meetings between the lead author and research coder confirmed appropriate sorting of the initial 20 articles and operational definitions for the 7 I+PSE components. Notes were written in the Mendeley annotations function for each article that was related to PSE and HEAL, providing the rationale for their folder assignment.

For the final review phase, 2 researchers were trained to independently examine the full text of articles describing PSE HEAL initiatives to determine if they included implementation and evaluation activities for any of the 7 I+PSE components. This training consisted of 2 coders and the lead researcher (L.C.S.) reviewing and coding the same 5 articles individually and then comparing their results. If any disagreements were noted, activities and components were discussed to determine final categorization. No reliability testing was done. For the remainder of the articles, coders noted any description of the 7 I+PSE components, whether or not specific activities were evaluated, and what evaluation frameworks and methods, if any, were used. Over several meetings, the 2 coders reviewed their coding for each article and reached consensus for either inclusion or exclusion. In instances of uncertainty, they consulted the lead researcher for a final decision. Additional notes were made to provide the rationale for these decisions.

### Data extraction

One coder extracted and entered data into a results table (Table 2). A second coder compared the articles’ content with table entries to confirm the accuracy of all content. The lead author reviewed all table content for consistency of descriptions. This table included the last name of the first author and publication date, funder(s) of the research described in the article, name and purpose of the initiative, study setting, and length of study. The table also indicated which of the 7 components of I+PSE were addressed in intervention activities, which were evaluated and how, accomplishments and challenges noted by the article authors, and comments from our coders on the extent of evaluations. This approach is an appropriate strategy for mapping reviews, rather than applying a more formal quality assessment tool (eg, Cochrane) (17). We used quantitative counts and qualitative content analysis strategies to summarize data and reveal themes as recommended by Miles et al (73). Themes from each column (eg, funding source, I+PSE components described) were inductively determined by first reviewing the content and subsequently creating categories. The same 2 researchers who entered and confirmed these data and the lead researcher determined the themes and categories together over several meetings. Counts were then generated for each category and summarized in narrative, table, or figure format. We did not attempt a meta-analysis because of the heterogeneity of initiatives and measured outcomes.

**Table 2:** Characteristics of Healthy Eating and Active Living Initiatives Described in Reviewed Studies (N = 52) That Used Policy, Systems, and Environmental Approaches^a^

Author, year (reference)	Funding source	Initiative name; purpose(s)	Setting	Length	I+PSE Framework components aligned with evaluation strategies a	Authors’ identified accomplishments and challenges; coder comments
Abildso, 2019 (19)	CDC Community Transformation Grant	Change the Future West Virginia; evaluate adoption and reach of nutrition-based PSE in food desert	638 Schools, 120 farmers markets, 47 retail food outlets	3 years	Online survey, (weak) [3]; Online survey, weak [4]; documentation, online survey [5]; online survey [6]. Evaluation methods: online survey of participating officials in schools, farmers markets, and outlets; documentation: copies of signed agreements with farmers markets and retail food outlets. RE-AIM Framework used	Accomplishments: Schools in 48 of 55 counties implemented farm-to-school activities; 2 of 3 farmers markets signed collaboration agreements; hiring of personnel with trust and connections was valuable for improving process; changes were easier in local grocery stores than in national stores. Challenges: Funding ended abruptly, and many objectives were not sustained. Lack of resource availability limited progress. Coder comments: Trainings and local connections (personnel-related implementation) important but give minimal details on how this was done.
Agner, 2020 (20)	CDC and Robert Wood Johnson Foundation	Healthy Hawai‘i Initiative (HHI); statewide effort to prevent and control chronic disease, extend and increase the quality of Hawaiians' lives, and address health disparity	Private and nonprofit organizations, schools, general public	Ongoing since 2000	10 In-depth, semi-structured interviews [4, 5, 6, 7]. Evaluation methods: 10 in-depth, semi-structured interviews with key informants and systematic literature review of HHI reports and articles. Culture of Health Action Framework; 1) creating health values, 2) cross-sectoral collaboration, 3) healthier communities, 4) strengthening health services and systems	Accomplishments: HHI has capitalized on relationship building, data sharing, and storytelling to encourage a shared value of health among lawmakers, efforts led to development of health policy champions; deemed overall a very successful program. Challenges: Cultural differences sometimes led to implementation pushback. Coder comments: Details of how triangulation was conducted or supports results is unclear.
Arriola, 2017 (21)	CDC funded Emory Prevention Research Center’s Cancer Prevention and Control Research Network to provide mini grants	Prevention Strategies that Work; measured congregants' perceptions of healthfulness of church environment, policies, and social support, and their physical activity and dietary behaviors in and out of church	6 Faith-based organizations in Georgia	12 months	No evaluation [3]; pre and post survey [5]; pre and post survey [6]. Evaluation methods: pre and post surveys administered to church members: pre (baseline) and post 1 year after	Accomplishments: increase in perceived healthy foods served at church associated with overall healthy foods eaten. Challenges: No significant relationship between changes in church physical activity environment and general physical activity behavior; longevity/ sustainability limited. Coder comments: measured “intention to use” for physical activity instead of actual behavior
Askelson, 2019 (22)	USDA funded Iowa Department of Education Team Nutrition program	[No named initiative]; describe implementation and results of lunchroom intervention using principles of behavioral economics	6 rural middle schools in Iowa	1 school year	Online survey [2]; semi-structured interview [3, 4]; lunchroom assessment, production records, semi-structured interview [6]. Evaluation methods: Online pre- and post-surveys assessed students’, parents’, and food service staff’s perceptions of the lunchroom; semi-structured telephone interviews to assess the experiences and perceptions of food service directors; lunchroom assessment tool assessed environment by measuring criteria during lunch period walk-throughs; production measuring lunch staff ordering/ preparation of healthy foods	Accomplishments: increased lunchroom assessment scores for 5 of 6 middle schools; 4 schools increased servings of healthy food; directors reported intervention as feasible long-term and well received; improved communication with students. Challenges: consumption not measured, no measure of staff and student interaction or education or how relationships between the two improved. Coder comments: production records not reflecting serving; lunchroom assessment not quantitative
Balis, 2019 (23)	SNAP-ED, EFNEP, University of Wyoming Extension	Take the Stairs, Wyoming!; increase physical activity in workplaces using PSE; implementation using posters encouraging stairway use	32 Wyoming businesses and organizations with elevators	6 months	Opportunistic interviews and site observations [6]. Evaluation methods: interviews with Extension Service health educators and observations of businesses and organizations	Accomplishments: posters widely adopted and implemented. Challenges: capturing reach, effectiveness, and maintenance was challenging because health educators found evaluation burdensome; therefore, difficult to tell whether posters were effective at increasing physical activity levels; lack of data collection adherence by health educators. Coder comments: limited evaluation.
Berman, 2018 (24)	J.R. Albert Foundation, Kansas Health Foundation, and Health Care Foundation of Greater Kansas City	Healthy Lifestyles Initiative; increase healthy eating and physical activity, and reduce obesity and disparities through PSE approaches	Local children's hospital, 218 community partners and 170 community organizations (schools, childcare providers, health care providers, businesses, nonprofit community organizations, government organizations)	1 year	Online survey [1]; focus groups and online survey [2]; online survey [3]; weak online survey [4]; online survey [5]. Evaluation methods: online survey emailed to partners, self-reporting initiative implementation, focus group to determine what health messaging would be the best/ most desired in targeted settings.	Accomplishments: educational handouts and posters most commonly used materials; partnerships and reviewing wellness policies most common activities; positive association between making an action plan and number of implementation strategies with activity implementation and material use. Challenges: partners reported wanting increased support; progress/ implementation slow because of lack of resources, communication, and need for additional materials and trainings (although receiving materials was not associated with material usage). Coder comments: minimal information on how self-reporting was standardized
Bunnell, 2012 (1)	CDC, US Department of Health and Human Services, county health departments	Communities Putting Prevention to Work; develop PSE plans for decreasing obesity through nutrition and physical activity, tobacco use, and secondhand smoke exposure	50 Communities (14 large cities, 12 urban areas, 21 small cities and rural counties, and 3 tribes in 32 states and District of Columbia)	2 years	No evaluation [3, 4]; milestones and action plans [5, 6]. Evaluation methods: review of action plans and outcome objectives, and quarterly reporting on outcome objectives and milestones	Accomplishments: more than one-third of communities advanced their obesity and tobacco-use strategies within 12 months; tobacco interventions had higher population reach than obesity interventions. Challenges: Implementation varied significantly across interventions. Reach was limited (35%–50%) for obesity programming. Coder comments: Evaluation plan discussed in minimal detail; vague and difficult to follow.
Castillo, 2019 (25)	CDC	Working on Wellness; PSE intervention for expanding bicycle infrastructure and opportunities for physical activity	General population (860,861 residents) of Hidalgo County, Texas	3 years	No evaluation [2, 3, 4]; no evaluation described but stated that it occurred [5, 6]. Evaluation methods: Baseline needs assessment of built environment; no further evaluation strategies described	Accomplishments: More than 5 miles of bike lanes installed; bike safety and group rides program initiated; Bicycle Friendly Business Program (57 bicycle racks installed) implemented; 9 of 10 bike trail plans implemented). Challenges: sustainability questioned; partner involvement not fully sustained. Coder comments: No measure on how residents’ use, health, behavior changed, only that access increased. No details on how reporting was facilitated.
Cheadle, 2010, Cheadle, 2012 (26,27)	Kaiser Permanente Northern California Community Benefit Program	Northern California initiative (Healthy Eating, Active Living –Community Health Initiative); to promote population-level improvements in intermediate outcomes (physical activity levels, proportion eating healthy diet) and long-term improvements in related health outcomes	3 largely ethnic minority communities with populations of 37,000–52,000; stakeholders were health care sites, worksites, neighborhoods, schools, food stores, and restaurants	5 years	Telephone survey of adults, school-based survey of youth, fitness test, height and weight measured [1]; interview and photovoice [2, 4]; no evaluation [3, 7]; DOCC; Photovoice [5]; DOCC; Photovoice [6]. Evaluation methods: telephone survey of members accessing Kaiser Permanente health services, pre/post surveys of youth in fitness program measuring physical activity and nutrition, pre/post fitness test of youth, height and weight measurements taken with a few participants, DOCC database tracked reach (number exposed and number affected) to quantify implementation, key stakeholder interviews and Photovoice to gather community opinion and perspective on initiative	Accomplishments: 76 Strategies across 3 communities, high dose interventions to increase youth physical activity, results of youth survey inconclusive. Challenges: Lack of long-term effects due to stakeholder, student, and patient turnover; telephone survey response rates too low to provide adequate information. Coder comments: measuring youth population change was resource intensive; lack of longitudinal measures; I+PSE 5 and 6 potentially sustainable.
Cheadle, 2016 (28)	Department of Health and Human Services, CDC Community Transformation Grants, and Partnership to Improve Community Health	King County Communities Putting Prevention to Work; PSE changes to reduce obesity and tobacco use	Schools, local government, community organizations (supported by public health department) across King County, Washington. Consisted of 7 low-income communities (652,000 residents)	24 months	Interviews [2]; no evaluation [3, 7]; key documents, meeting minutes and attendance, online surveys, tracked advocacy efforts [4]; interviews [5]; interviews, environmental assessments [6]. Evaluation methods: Residents surveyed online, interviews with key stakeholders, observed activities, assessed environment, tracked joint advocacy efforts, reviewed key documents (ie, project planning, action summaries), meeting attendance, estimated number of residents reached	Accomplishments: 22 of 24 strategies achieved significant progress; dyad of technical assistance provider plus champion were key to success; unable to gain joint use agreement. Challenges: More information/ discussion necessary on long-term policy. Coder comments: Environmental assessment seems weak, not standardized
Coleman, 2012 (29)	USDA National Research Initiative Award	Healthy Options for Nutrition Environments in Schools; randomized group trial of schools that implemented school nutrition policy and environmental changes to reduce unhealthy foods and beverages on campus, develop nutrition services as the main provider for healthy eating, and promote staff modeling of healthy eating	1 Low-income school district with 6 elementary schools and 2 middle schools	3 years	Interviews, height and weight measured [2]; no evaluation [3]; policy document review, environmental assessment, interviews (parent, student, teacher), fundraising results [5]; environmental assessment, count outside beverages per child per week in cafeteria and at recess [6]. Evaluation methods: Interviews of parents, students, and teachers regarding implementation and opinions of PSE changes, self-reported student height and weight, number of outside foods and beverages counted in observation, environment assessment by counting products in trash bins after lunch period, semi-structured interviews with school district administrators and principals, review of policy documents, funding results (money raised and attendance). Model/framework: Plan-Do-Study-Act, Institute for Healthcare Improvement's rapid improvement process model	Accomplishments: Outside food and beverages per child per week decreased for intervention schools and increased for control schools over time (especially for unhealthy items); changes in rates of obesity were similar in both intervention and control schools. Challenges: Low participant buy-in; unsure whether due to resources or engagement. Coder comments: No exploration of why no significant changes seen between two conditions.
Cranney, 2021 (30)	New South Wales (NSW) Ministry of Health, Prevention Research Collaboration	The Healthy Food and Drink in NSW Health Facilities for Staff and Visitors; aims to increase availability and promotion of healthy food and drink options in food outlets in NSW Health facilities	15 NSW Local Health Districts and 3 Specialty Health Networks; 26 public hospitals and health facilities and 691 food outlets (eg, kiosks, vending machines, coffee shops)	1 year	Audit [5]; audit and survey [6]. Evaluation methods: Audit of random samples (environment and adherence to policy); consumer intercept survey	Accomplishments: Proportion of outlets that removed sugary beverages increased from 58.0% to 96.3%; majority of outlets supported removal, with nearly half reporting it would improve people's health. Challenges: Baseline not obtained for nearly 2/3 of implementation locations; baseline data collection seemed to “motivate” changes. Coder comments: Evaluation done only in a portion of locations (both audit and intercept survey). No measure of how consumers engaged with policy and environmental changes. Strength: Audit collection tool was standardized and auditors were trained.
Eisenberg, 2021 (31)	CDC	Reaching People with Disabilities Through Healthy Communities; infuse disability inclusion into PSE changes promoting healthy living (help improve access to healthy choices for community residents with disabilities)	10 Communities across Iowa, Montana, New York, Ohio, and Oregon	2-1/2 years	The Community Health Inclusion Index (CHII) assessments [5, 6]; walkability audits; walkability audits, CHII assessments [7]. Evaluation methods: CHII assessments; walkability audits; CHII questions on level of inclusion; recorded data on standardized spread sheet that updated quarterly, content analysis. Framework: Guidelines, Recommendations, Adaptations Including Disability (GRAIDs)	Accomplishments: Implemented 507 inclusive PSEs, 466 were environmental changes, 25 systems changes, and 16 policy changes. Strong internal validity. Challenges: Inability to note intersect. Coder comments: Insufficient description of content in assessment; assumed from reported evidence.
Escaron, 2019 (32)	CDC	[No name noted]; promote healthy eating throughout the school day and in after-school programs	19 School and after-school programs (Boys and Girls Club and YMCA) in southeast Los Angeles, California	4 years	The Healthy Afterschool Activity and Nutrition Documentation Instrument (HAAND) interviews [3]; program records, the Wellness School Assessment Tool (WellSAT) 2.0 [5]; HAAND interview, HAAND pre- and post- assessment, WellSAT 2.0 [6]; no evaluation [7]. Evaluation methods: Interviews with program staff regarding effectiveness of trainings, their opinions of the HAAND-based guidelines, HAAND assessment pre–post with rubric to evaluate policy, program records regarding trainings/ engagement, WellSat 2.0 to evaluate quality of policy/ implementation. Evaluated via RE-AIM framework	Accomplishments: Reached 43.5% of priority student population. Improvement in wellness policy quality and after-school practices pre- to post-intervention. Challenges: Pushback and lack of communication from some district administration; limited implementation. No follow-up evaluation. Coder comments: Very brief mention of training; weak measure of student engagement.
Feyerherm, 2014 (33)	CDC Communities Putting Prevention to Work	Partners for a Healthy City; collaborations with local organizations to implement policies to promote healthy eating and physical activity; develop partner engagement kit, recruit community trainers, and provide technical assistance	346 Organizations: faith-based organizations, businesses, physician offices, and agencies in Douglas County, Nebraska	2 years	No evaluation [2, 3, 4, 6]; online assessment tool, number of signed letters of intent [5]. Evaluation methods: Online assessment tool for baseline of policy quality, follow-up assessment to evaluate new policy implementation, number of letters of intent to implement at least 1 policy	Accomplishments: 92% of organizations implemented 1 new policy or expanded a current policy; 952 policy changes total; careful selection of community trainers, wide range of policies, alignment of initiative with organization initiatives, and incentives contributed to success. Challenges: No evaluation on whether or not 1 policy change resulted in many more. Coder comments: Letters of intent weak form of evaluation.
Garcia, 2017; Garcia 2018 (34,35)	CDC, American Heart Association	Accelerating National Community Health Outcomes Through Reinforcing Partnerships Program; PSE interventions to increase healthy food options in vending machines	8 Communities, state capitol, city building, community organization, vending machines	16 months	No evaluation [1, 2, 3, 4]; assessment [5, 6]. Evaluation methods: Nutrition Environment Measures survey vending assessment to evaluate environment at baseline and post initiative	Accomplishments: 63% of vending machines had healthier food or beverages at follow-up. Challenges: Pushback from community about vending changes and delays in follow-up because of vending contracts and other priorities; institutional and community buy-in was important for implementation; 8 communities assessed baseline, but only 3 communities assessed follow-up. Coder comments: 1,2,3,4 not evaluated. Question whether environment assessment really evaluated I+PSE component 5 (policy)
Garney, 2018 (36)	CDC, American Heart Association	Accelerating National Community Health Outcomes Through Reinforcing Partnerships Program; Multiple case study design to implement PSE interventions to increase access to healthy foods and beverages, physical activity, and smoke-free environments	6 nonprofits (and surrounding communities) in 6 states	Began in May 2015 [length of time unknown]	Action plan assessment, interviews [2]. Evaluation methods: Interviews with program staff and community partners; Quality of Action Plan Assessment Framework, Consolidated Framework for Implementation Research, State Plan Index Tool	Accomplishments: Implementation-ready communities felt they were most successful in community engagement, even though they were also able to successfully implement PSE interventions. Challenges: Lack of time, lack of follow-up, and difficulty accessing key stakeholders impeded capacity-building communities’ progress. Both types ultimately received community support; just took longer in capacity-building communities. Capacity-building sites laid the groundwork for change but struggled to achieve tangible outcomes. Coder comments: Interviews implied I+PSE component 3 happened. No measure that policy I+PSE component 5 was implemented.
Garney, 2020 (37)	CDC	Accelerating National Community Health Outcomes through Reinforcing Partnerships Program; preventing chronic disease by targeting cardiovascular disease risk factors (access to fruits and vegetables, physical activity, and smoke-free environments)	15 community-based cardiovascular disease prevention partnership networks in the Northeast, South, Midwest, and Western US. 39% of the communities had a poverty rate below the state and/or federal rates	14 months	Interorganizational Network (ION) survey and partner interview [4]. Nutrition Environment Survey [6]. Evaluation methods: ION survey regarding collaboration, interview with partners involved, survey regarding nutrition environment	Accomplishments: 73% of communities met partnership goals. More equal partnerships were correlated with more success than hierarchical ones. Triangulation with qualitative data. Challenges: Limited timeline; low response rate in some networks; level of involvement required unclear. Coder comments: Subjective data interpretation; complex analysis not available for all initiatives. Mentions evaluation of I+PSE component 6 but 6 wasn't clearly stated as a goal.
Garvin, 2013 (38)	Virginia Department of Health, Consortium for Infant and Child Health	Business Case for Breastfeeding program; lactation support program to eliminate breastfeeding barriers by encouraging changes in policy and business environment	20 businesses in southeastern Virginia	14 months	No evaluation [1]; lactation assessment form (LAF) ordinal rating and follow-up questionnaire by phone (weak; indirect) [2]; no evaluation [4]; LAF ordinal rating and follow-up questionnaire by phone [5,6]. Evaluation methods: LAF ordinal rating evaluated environment and policy with rubric-based scale, follow-up questionnaire by phone of businesses measuring engagement in environment. Transtheoretical Model of Behavior Change adapted to assess stage of organizational change. CDC tool for measuring community-level PSE change adapted to assess worksite policy and environmental changes	Accomplishments: 20 businesses educated, 17 engaged, 15 completed LAF questionnaires; positive engagement and implementation of the Business Case for Breastfeeding toolkit. Challenges: Only implemented in health care businesses. Coder comments: Appears some sites opted out midway because of minimal support/ communication.
Giles, 2012 (39)	CDC Nutrition and Obesity Policy Research and Evaluation Network and Prevention Research Centers Program, Donald and Sue Pritzker Nutrition and Fitness Initiative, and Robert Wood Johnson Foundation	Out of School Nutrition and Physical Activity Initiative; implement low-cost strategies to provide water after school; increase consumption of water by elementary school–aged children during after-school snack time; increase overall youth health	20 Randomly selected after-school programs in the Boston, Massachusetts area	1 School year	No evaluation [2, 3, 4]; observer records [5,6]. Evaluation methods: Observer records from what was served during after-school programs (records noting times per day served, volume by ounce served, frequency served, calories in other beverages served; related caloric consumption calculations) based on social–ecological model and a community-based participatory research approach	Accomplishments: Water served to children increased in programs receiving intervention resources, calorie consumption decreased compared with nonintervention programs. Challenges: 1 school year, only elementary school–aged children. Coder comments: Minimal evaluation of components, no longevity assessment.
Gollub, 2014 (40)	CDC	Louisiana's Tobacco Control Program's Putting Prevention to Work; engage school communities to create an environment that promotes healthy eating, physical activity, and tobacco-free living	27 Louisiana Public Schools	1 School year	Survey, social media progress report, telephone calls [2]; learnings survey, training sessions report visits, best practice reporting [3]; survey (weak), best practice reporting (weak), overall no evaluation [5]. Evaluation methods: Survey of youth behavior/ beliefs, social media campaign/ content report, telephone calls regarding opinion of social media campaigns, regular on-site learnings survey of state team members on lessons learned during planning and implementation processes, reports on what happened during training sessions, monitoring/ documentation of best practices via site reporters	Accomplishments: Environmental changes (eg, physical activity breaks, healthier vending options, tobacco-free campuses) were adopted. Challenges: Only 25 of 27 schools finished. Coder comments: Survey content/ purpose ambiguous, weak evaluation of I+PSE category 5; very focused on I+PSE category 3
Hardison-Moody, 2011 (41)	Kate B. Reynolds Charitable Trust of Winston-Salem	Faithful Families Eating Smart and Moving More; promote health of low-income faith communities through 9-session EFNEP-led educational series on good nutrition and physical activity; promoted health with environmental and policy changes to support learning	44 Faith communities in North Carolina	9 Weeks	Member health assessment [1]; no evaluation [3]; 172 policy and environmental changes enacted [5,6]. Evaluation methods: Member health assessments pre and post, reporting (very vague)	Accomplishments: Communities enacted 172 policy and environmental changes; behavioral changes noted. Challenges: Difficulty in communication between Extension officials and leaders in the faith-based sector; differences in communication/ implementation. Coder comments: No reporting on how policy/ environmental change data presented/ obtained. Evaluation methods not discussed in detail
Hardison-Moody, 2020 (42)	USDA Regional Center for Nutrition Education and Obesity Prevention, Southern Region	Faithful Families; adoption of PSE changes to support healthy eating and physical activity in faith communities through coordinated effort between EFNEP and SNAP-Ed	13 Faith communities in Tennessee, Arkansas, and Florida	1 Year	No evaluation [1,3]; site reports [5,6]. Evaluation methods: Faith community assessment, site reports on environmental changes promoting healthy choices from site visits, and annual reporting.	Accomplishments: 34 PSE changes implemented; affected 11 faith communities with 4,810 members across sites. Challenges: Small sample; no long-term effects noted; very difficult to recruit lay leaders; reach could have potentially been greater. Coder comments: Reporting vague, audit tool used not described in detail
Hearst, 2018; Hearst, 2019 (43,44)	NHLBI/NIH	BreakFAST; After school breakfast policy and environment to change adolescent beliefs of barriers and benefits of breakfast	16 Rural public high schools in Minnesota	2 years	Student surveys [1]; no evaluation [2, 5, 6]. Evaluation methods: Written student survey asking how many days a week they ate breakfast	Accomplishments: Changes to school policy and environment led to “Grab and Go” and “2nd Chance” breakfast options and marketing strategy; intervention versus control students reported significant reduction in perceived barriers to school breakfast. Challenges: No intervention difference in beliefs surrounding importance of breakfast. Coder comments: Applied group randomized design to provide strong evidence
Holston, 2020 (45)	CDC	Healthy Communities; implement PSE strategies supporting education to promote healthy behavior change and overcome barriers	3 Rural parishes in Louisiana with adult obesity rates higher than 40%	3 Years	No evaluation [3, 4, 6]. Evaluation methods: Simply stated initiatives, unclear how results obtained/reported	Accomplishments: Interventions varied by parish but included Complete Streets implementation plans, healthy retail initiatives, play space improvements, downtown beautification projects, and Smarter Lunchrooms. Extension office still has member advocating for program. Challenges: Progress was slow; instability between management/ leadership at parishes made implementation and reporting difficult/inconsistent. Prevention changes not used/not sustainable. Coder comments: Unable to understand purpose of technical assistance/education from Extension office (I+PSE component 3)
Honeycutt, 2012 (46)	CDC and NCI/NIH	Nutrition Programs that Work; mini-grants and technical assistance to disseminate evidence-based programs, understand how the project worked in different settings, and generate recommendations for future programming and evaluation	7 Churches and worksites in rural, southwest Georgia	18 Months	Activity forms and interviews (weak) [1]; no evaluation [3]; activity forms, interviews, and focus groups [5, 6]. Evaluation methods: Program records (not specified); activity forms completed by grantees; interviews with organizational leaders about program implementation and focus groups that collected information about their activities during telephone calls and site visits. Framework: RE-AIM framework	Accomplishments: All 7 sites conducted at least 6 of 8 core elements including at least 1 food-related policy or environmental change; program reach varied widely across sites and core elements. Challenges: Sites intended to discontinue some aspects of program; some participants unwilling to provide data; only program leaders at worksites. No follow-up data taken, only data during intervention. Coder comments: Pushback in adoption of program resulting from paperwork required for evaluation; no specification of what program records evaluated
Jernigan, 2018 (47)	NHLBI/NIH	Tribal Health and Resilience in Vulnerable Environments [THRIVE]; increase vegetable and fruit intake among Native Americans living within the Chickasaw Nation and Choctaw Nation of Oklahoma	20 Convenience stores owned or operated by Chickasaw Nation and the Choctaw Nation of Oklahoma	5 Years	Sales data [1, 6]; no evaluation [2, 4]. Evaluation methods: Sales data	Accomplishments: Increased sales of fruits and vegetables; evaluation in progress. Challenges: Extensive cost and staff support; not generalizable to all Indigenous communities. Difficult to determine if intervention eliminated disparities. Coder comments: Sales data only (and limited) measure of promotion, store placement, owner supply, and consumer behavior
Kao, 2018 (48)	Kaiser Permanente Community Health Initiative	Healthy Eating Active Living project; workshop and technical assistance to develop policy for physical activity and other healthy behaviors (part of larger community collaborative)	17 Licensed family childcare homes in Northern California	3 Years	No evaluation [2]; pre and post observations, questionnaires, physical activity logs [5, 6]. Evaluation methods: Pre- and post-intervention evaluation without a control group; pre and post observations, self-assessment questionnaires, and physical activity logs	Accomplishments: Providers increased number of activities, improved screen time practices, and made improvements to the physical activity environment. Challenges: Variability in reporting, low implementation of physical activity promotion material. No significant change in size of play space or total physical activity. Coder comments: Trained data collector conducted objective observations of physical activity environment to resolve subjectivity in reporting
Kegler, 2015 (49)	CDC and Mississippi State Department of Health	[No name noted]; Community-based PSE initiative to support cardiovascular disease and stroke prevention. Guided by Advisory Council and implemented by the Mississippi State Department of Health in collaboration with local community partners	7 Mississippi Delta counties across health, faith, education, worksite, and community/city government sectors	3 Years	Grantee interview, grantee progress report, health council member survey [2]; grantee progress report [3]; health council survey [4]; grantee progress report, organizational survey, health council survey, church PSE survey [5]; grantee progress report, church PSE survey [6]; no evaluation [7]. Evaluation methods: Grantee interview to evaluate intervention; grantee progress reported/documented intervention progress survey with health council to determine engagement/ progress; organizational survey to evaluate progress and (weakly) quality of partnerships; church survey to evaluate PSE changes in each church	Accomplishments: Increased PSEs seen across all sectors. Support increased access to physical activity opportunities, healthy food and beverage options, quality health care, and reduced exposure to tobacco. Challenges: Timeframe and funding limited scope of intervention and evaluation. Challenging to collect data, evaluate/ modify training aspect. Lack of baseline data. PSEs not adopted equally across all sectors attributed to lack of cultural modification for each sector. Coder comments: Lack of deep evaluation/modification in I+PSE components 2, 3, 4
Kelly, 2021 (50)	Colorado Department of Public Health and Environment’s Cancer, Cardiovascular, and Pulmonary Disease	The Colorado Department of Public Health and Environment’s Cancer, Cardiovascular, and Pulmonary Disease grant program; implement policy and environmental strategies that support HEAL. Coalitions prioritized built environment to support active living and healthy food and beverage strategies	6 Coalitions in Colorado representing 5 communities	3 Years	No evaluation [3, 4]; semiannual report and follow-up calls [5, 6, 7]. Evaluation methods: Semiannual reporting by coalitions about their communities’ progress. Follow-up call with coalition leaders to validate reporting.	Accomplishments: Built environment coalitions implemented change at 61 sites with 16 policies and plans and 44 environmental changes. Healthy food and beverage coalitions implemented changes at 66 sites by passing 31 policies and plans and 44 environmental and practice changes. Challenges: Self-reports by coalitions; inconsistent methodology across sites and potentially inflated numbers. Implementers reported long and slow progress not sustainable for the long term. Coder comments: Very little discussion of how reporting occurred
Leser, 2021 (51)	CDC’s Healthy Communities Program	Action Communities for Health, Innovation and Environmental change; establish partnerships between national and local organizations to promote and implement structural changes in local communities across the US to address risk factors for heart disease, stroke, diabetes, cancer, obesity, and arthritis	10 Community-based organizations in Ohio	1 Year	No evaluation [4]; survey and interview [5]. Evaluation methods: Survey of organization members using closed-end questions; an in-depth qualitative interview with organization leaders	Accomplishments: Policies either fully (66%) or partially (31%) implemented. Participants (97%) reported that they somewhat/strongly agreed that the adopted policies would be implemented in the future. Challenges: Some staff pushback in health-related policy. Coder comments: No description of how support was provided to organizations (how did initiative support aim). Description alludes to collaboration between different levels of large and small organizations, but not in detail
Long, 2018 (52)	CDC, NIH	Sodium Reduction in Communities Program; reduce sodium intake through food service guidelines, procurement and food preparation practices, and environmental strategies	30 Public schools and 5 community meal programs in Northwest Arkansas	1 Year	No evaluation [2, 3, 4]; procurement records, food production records [5]; observation and documentation of food preparation [6]. Evaluation methods: Schools: procurement records, food production records, number of people served, menu item nutrient reports, implementation records, point-of-service, and sodium data. Community meals: implementation records, procurement records, daily menus, daily counts of people served, observation and documentation of food preparation, point-of-service, and sodium data	Accomplishments: Mean sodium levels per lunch diner decreased 11.2% in schools and 16.6% in community meal programs. Challenges: Implementation time; staff-intensive. No control group. Staff ability to program, advertise, and prepare food delayed or lacking. Staff lacked monetary incentive to support policy. Coder comments: Little focus on I+PSE categories 2, 3, 4. Not sure how success or limitation of these influenced results
Long, 2019 (53)	CDC Racial and Ethnic Approaches to Community Health [REACH] grant, National Institute of General Medical Sciences grant, and NIH Translational Research Institute grant	REACH project; improve food quality and increase distribution of fruits and vegetables at food pantries for 1,500 Pacific Islander and Hispanic clients	3 Food pantries in Arkansas	13 Months	Food pantry bag audits and client surveys [1]; no evaluation [3, 4, 5, 6]. Evaluation methods: Food pantry bag audits assessing nutritional content, survey of client opinions regarding PSE changes	Accomplishments: Modest increase in fresh fruit and vegetable servings; reduction in sodium per 2,000 calories distributed. Challenges: No control group. No determination of longevity of project. Difficult to generalize to other communities. Coder comments: Not sustainable or generalizable without policy evaluation.
Martin, 2009 (54)	Master Settlement (Funds from master state tobacco settlement)	Healthy Maine Partnerships; reduce tobacco use, increase physical activity, and improve nutrition through local policy and environmental change	Schools, worksites, hospitals, municipalities, colleges, restaurants, food pantries across Maine	3 Years	No evaluation [3]; narrative reports [4]; outcome survey [5, 6, 7]. Evaluation methods: Outcome survey from 31 local partnership directors and school health coordinators, and Five-Year Review (narrative) measured activities and outcomes for all components	Accomplishments: 4,600+ policy or environmental changes reported; tobacco use policies represent most changes implemented. Extensive training and technical assistance improved skills of public health workforce. Challenges: High staff turnover; trainings not fully redone. Coder comments: Limited/ minimal results reported
McGladrey, 2019 (55)	CDC	[No name noted]; enhance collective impact of systems-level physical activity promotion programming through a multisectoral approach	Clinton County Kentucky Extension Office	3 Years	Survey after training on coalition content [3]; relationship survey [4]; environment and program survey [6]. Evaluation methods: Survey after trainings to assess understanding and opinions on content, survey of partners to assess quality of assistance and partnership (vague), survey regarding environment and program changes	Accomplishments: Extension-led coalition accomplished the 6 essential functions of a backbone support organization by identifying obesity as a critical local issue. Infrastructure for future initiatives well-established. Relationships and program deemed sustainable. Challenges: Coalition-building difficult in rural areas because of geographic isolation and deficits in infrastructure, public transportation, health care providers, and funding. Coder comments: Overall description of evaluation very ambiguous and confusing. Discussion of triangulation, but unclear what was achieved
Molitor, 2020 (56)	SNAP/USDA	SNAP-Ed; evaluation to correlate number of SNAP-related PSE changes to dietary behavior and diet quality of caregivers	2,222 Household caregivers in California (58 counties)	1 Year	Dietary recall assessment [1]; activities entered into SNAP-Ed program, evaluation, and reporting systems [6, 7]. Evaluation methods: 24-hr dietary recall of caregivers’ assessment by telephone compared by census tract where PSE and direct education occurred	Accomplishments: PSE reach predicted decreased intake of sugar-sweetened beverages and added sugar, and increased Healthy Eating Index, regardless of race and/or ethnicity, age, or reach of direct education. Challenges: Self-reporting on behaviors/diets, minimal measure of diet quality, only number of PSE activities to which participants may have been exposed. Coder comments: Unable to tie diet quality to PSE change as latter was loosely evaluated
Murriel, 2020 (57)	CDC	High Obesity Program; implement PSE changes to support healthy eating and access to physical activity opportunities	Coalitions in 100 communities across 11 states (Alabama, Arkansas, Georgia, Indiana, Kentucky, Louisiana, North Carolina, South Dakota, Tennessee, Texas, West Virginia)	4 years	Annual report and performance evaluation (weak) [3]; annual report [6]; Evaluation methods: Annual reports from communities and performance evaluations from project evaluator	Accomplishments: Increased access to physical activity for nearly 1.6 million people. Challenges: Varying success across community types (urban vs rural). Limited ability for evaluation (brief, not entirely thorough). No standardized way of initiative implementation because of number and variety of populations. No evidence for sustainability. Coder comments: Minimal discussion of how reporting worked (Who? How?) or what classified/ operationalized a true “PSE change”
O'Hara-Tompkins, 2021 (58)	USDA (Extension services under Agriculture Improvement Act [Farm Bill])	[No name noted]; improve quality and quantity of healthful foods and opportunities for physical activity provided to children, provider-child interactions, and potential for PSE changes related to healthful eating and physical activity	25 rural early care and education settings in 3 West Virginia counties	2 years	Online and print self-assessments [1, 5, 6]; no evaluation [3]. Evaluation methods: Online and print provider self-assessment for policy changes and personal engagement (weak)	Accomplishments: 148 PSE changes reaching approximately 450 young children. Challenges: Childhood obesity not investigated long-term. Unsure of longevity of changes. Difficulty with some providers not willing to/ not knowing how to uphold PSE changes. Pushback/challenges reported in providing physical environments. Coder comments: Very few details on what occurred during trainings/outcome of trainings. Very few details on how PSE changes were defined, reported, and recorded
Ratigan, 2017 (59)	CDC Putting Prevention to Work through County Health Agencies	Farmers' Market Fresh Fund Incentive Program; increase access to fresh produce for participants using US government assistance	4 farmers markets in San Diego, California	18 Months	Survey and market attendance [1]; no evaluation [4, 6, 7]. Evaluation methods: Survey (baseline, and in 3-month intervals) reporting market goer diet, purchasing behavior, opinion of program, and market attendance reports.	Accomplishments: Greater number of market visits associated with increased fruit and vegetable consumption and perception of higher diet quality. Odds of increasing fruit and vegetable consumption increased by 2% per month. Challenges: Program discontinued after 6 months. Lack of sustainability. Coder comments: Vague description of how implementation occurred.
Robles, 2019 (60)	SNAP-Ed/USDA	Small Corner Store Project; promote and support corner stores to encourage patron selection of healthier food items	13 Corner stores in Los Angeles County, California	3 Years	No evaluation [3]; corner store conversion (CSC) staff interview (weak) [4]; CSC staff interview [5]; landscape analysis Communities of Excellence in Nutrition, Physical Activity, and Obesity Prevention (CX3), environmental scans, patron interview [6]. Evaluation methods: Landscape analysis based on information from CX3 to determine quality of environment to support PSE changes, CSC staff informal interview (weak evaluation method), interview with corner store patrons to determine quality of store environment changes	Accomplishments: 6 stores received both baseline and follow-up assessment with 34% improvement in CX3 scores. Challenges: For some stores inadequate food distribution or lack of capital improvement infrastructure (eg, refrigeration) led to adoption pushback/inability; limitations in staffing led to pushback. Coder comments: No measure of I+PSE category 3; CSC staff evaluation was weak measure of I+PSE category 4
Robles, 2019 (61)	SNAP-Ed/USDA	Nutrition Education and Obesity Prevention Faith-Based Project; implement PSE change interventions at church sites alongside usual delivery of health education	11 churches in Los Angeles County, California	3 years	Congregant survey [1]; congregant interviews (weak) [2]; key informant interview [5, 6]. Evaluation methods: Congregant survey (semi-structured) noted signage and engagement, key informant interview measured quality and quantity of policy and environment (pre and post)	Accomplishments: Greater interest in eating more fruits and vegetables (66%), choosing water over soda (69%), and becoming more physically active (59%). Challenges: Did not account for demographic variation among sites. Limited ability of staff to relate to/work with/train leaders of the faith-based sector; low buy-in of leaders. Limited infrastructure slowed process of implementation. Coder comments: Key informant interview potentially weak evaluation of I+PSW categories 5 and 6
Ryan-Ibarra, 2020 (62)	SNAP-Ed Southeast Region/USDA and NIH	[No name noted]; 1) assess relationship of direct education on healthy eating and shopping behaviors and 2) collect PSE change data related to nutrition supports	25 Implementing agencies in Alabama, Florida, Georgia, Kentucky, Mississippi, North Carolina, South Carolina, and Tennessee	1 Year	Pre–post survey [1]; no evaluation [2, 3, 4]; observation, interviews with key informants, repeated assessments or surveys, photographic evidence [5, 6]. Evaluation methods: SNAP-Ed Evaluation Framework	Accomplishments: Participants improved diet quality and food behaviors post intervention; 701 PSE changes reached 830,049 people (most were environmental and systems changes, 471 promotional efforts) Challenges: States used different education programs, surveys, and PSE strategies making combining evaluation content difficult. Coder comments: Application of evaluation framework, multistate, multiple sources of PSE evidence; no control group or matched/pair analysis; no evaluation for I+PSE categories 2–4
Saunders, 2019 (63)	CDC	Faith, Activity, and Nutrition; promote physical activity and healthy eating through church PSE change	54 Churches in South Carolina	1 Year	No evaluation [3]; telephone interviews; site visits; surveys [5]; data collectors blind visits and surveys [6]. Evaluation methods: Baseline and 12-month telephone interviews, data collectors’ visits 8 to 12 months into intervention, surveys of church members evaluating policy and environment changes	Accomplishments: Higher levels of implementation for physical activity opportunities, physical activity and healthy eating guidelines, physical activity and healthy eating messages, and physical activity and healthy eating pastor support in intervention versus control churches. Challenges: Evaluation limited by self-reported survey method. Pastor support/partnership varied. Coder comments: No evaluation for I+PSE category 3.
Schroeder, 2018 (64)	NHLBI/NIH	Project breakFAST; increase high schoolers' breakfast participation by increasing availability of school breakfast, marketing breakfast, and providing opportunities for positive interactions that encourage breakfast participation	16 Rural Minnesota schools	4 Years	Monitor and document best practices, regular on-site visits and phone calls [3]; no evaluation [5]. Evaluation methods: Monitoring and documentation of best practices in implementation based on meeting discussions, regular on-site visits and phone calls used as a means to evaluate quality of training.	Accomplishments: Intervention schools increased participation in school breakfast by 56%, comparison schools increased participation by 7%. Control (non-breakfast eating) group allowed for better evaluation. Challenges: Despite slow progress in implementing “grab and go” lunches, technical assistance, and training, eventual implementation was possible. Coder comments: No mention of sustainability/ longevity
Schwarte, 2010 (65)	California Endowment Fund	Central California Regional Obesity Prevention Program (CCROPP); promote safe places for physical activity, increase access to fresh fruits and vegetables, and support community and youth engagement in local and regional efforts to change nutrition and physical activity environments for obesity prevention	Health departments, community partners, and communities of 8 California counties (Fresno, Kern, Kings, Madera, Merced, Stanislaus, San Joaquin, and Tulare)	3 Years	No evaluation [2, 3, 4, 5, 7]	Accomplishments: CCROPP has made progress in changing nutrition and physical activity environments by mobilizing community members, engaging and influencing policy makers, and forming organizational partnerships. Challenges: Only sustained in 1 community. Coder comments: No description of evaluation methods; unsure how conclusions were reached
Schwartz, 2015 (66)	CDC	Ten Steps to Successful Breastfeeding Initiative; improve efforts for better breastfeeding outcomes	8 Health centers serving predominantly Latino and Native American communities in Washington State	1 Year	Follow-up self-assessment, final self-assessment [3]; Evaluation methods: Follow-up self-assessment 6 months later on training received/ how well adhered to training and steps; pre and final self-assessment	Accomplishments: Within 6 months clinics fully operationalized between 2 and 7 steps. Sustainability seems hopeful. Challenges: Short intervention time, difficulty standardizing expectation/definition of full implementation of each step. Coder comments: Intervention only consisted of guidance in how to implement steps; didn’t actually help facilitate
Seguin, 2014 (67)	NHLBI/NIH	Strong Women Change Club (SWCC); engage individuals to identify relevant community issues and facilitate an action plan to affect social, cultural, environmental, and political factors	7 Rural communities across the United States	1 Year	Interview [2], 5-point scale [5]; interview (weak) [3]; interview, 5-point scale [6]; Evaluation methods: 5-point scale to rate progress and community engagement, follow-up interviews coded for common themes	Accomplishments: Each SWCC had achieved at least 1 benchmark; majority completed ≥3. Challenges: Busy schedules, resistance to and slow pace of change. Club members sometimes lacked interest/engagement. Coder comments: Study was derived from a 10-year partnership of collaboration between university researchers and community health educators/leaders, but I+PSE category 4 is not evaluated and not a focus of this study.
Shin, 2015 (68)	Robert Wood Johnson Foundation	[Name not found]; Increase availability and selection of healthful foods through nutrition promotion and education by using point-of-purchase materials (posters and flyers in stores) and interactive sessions such as taste test and cooking demonstrations	14 Recreation centers and 21 corner stores in Baltimore city area of Maryland, targeted youth	8 Months	Post-intervention surveys and intervention exposure evaluation [1]; youth peer educator feedback (weak) [3]; Youth Impact Questionnaire, Post-Intervention Survey, Intervention Exposure Evaluation [6]; Evaluation methods: To facilitate recall, a booklet containing pictures of intervention materials and intervention corner stores was shown to respondents as a prompt. Post-intervention survey evaluating beliefs, knowledge, and environment; intervention exposure evaluation to measure number of times promotional/educational material seen and outcomes; youth peer educator feedback used to evaluate trainings (weak); youth impact questionnaire to evaluate noticed environment	Accomplishments: Reduction in body mass index (BMI) in intervention girls. Moderate reduction of BMI when healthful foods were not as available. Food-related outcome expectancies and individual knowledge increased. Challenges: Control not true control (some control participants exposed to intervention strategies); overall low engagement (more support and collaboration needed). Not sustainable. Coder comments: Policy not supporting changes, and no policy evaluation
Subica, 2016 (69)	Robert Wood Johnson Foundation	Communities Creating Healthy Environments Initiative; apply community organizing to combat childhood obesity–causing structural inequities in communities of color, primarily designed to increase children’s healthy food and safe recreational access	21 community-based organizations and tribal nations across the United States	3 years	No evaluation [1, 3, 5]; community kitchen; grant interviews, post-grant interviews; public records, expert examination, community kitchen [6]. Evaluation methods: Quarterly interviews with grant leaders about progress of implementation, interview with grant leader at end of initiative; public records verifying PSE “wins,” experts examining quality of policy and its implementation; community kitchen mentioned as major PSE development.	Accomplishments: Grantees achieved 72 policy wins across 6 domains: healthy food, recreational access, promoting access to quality health care, clean environments, affordable housing, and discrimination- and crime-free neighborhoods. Challenges: Ineffective evaluation of community engagement; sustainability unknown. Coder comments: Limited evaluation methods for I+PSE categories 1, 3, and 5
Tomayko, 2017 (70)	Wisconsin Partnership Program Grant, NIH (multiple sources)	Active Early 2.0; evaluate effectiveness of previous program and provider training, micro-grant support, and technical assistance to increase physical activity and related behaviors to overcome obesity	Public schools across Wisconsin (~500 children reached)	2 Years	No evaluation [3]; Environment and Policy Assessment and Observation (EPAO) instrument [5]; document review, observation, exit interview, observation [6]; Evaluation methods: EPAO instrument to asses quality of policy, observation of children's activity using Actical triaxial accelerometers (1 day); exit interview evaluating how policy has aligned/changed (success measure)	Accomplishments: Significant improvements in total nutrition score and several nutrition subscales as guided by policy; percentage of sites with written activity policies moderately increased. Challenges: High turnover of staff involved with initiative. Coder comments: No evaluation for I+PSE category 3; noted challenge
Trieu, 2018 (71)	National Health and Medical Research Council of Australia under the Global Alliance for Chronic Disease Hypertension Program	Monitoring and Action on Salt in Samoa; lower population salt intake comprising awareness campaigns, better community mobilization, and creation of policy and environmental changes	Awareness campaigns via TV, radio, events, newspaper, billboards, food industry, schools; 780 randomly selected participants aged 18–64 years in urban and rural regions of Samoa	18 Months	Post-intervention survey, stakeholder interview [1]; post-intervention survey [2, 6]; no evaluation [7]. Evaluation methods: Post intervention survey of participants, interview with stakeholders after implementation	Accomplishments: Awareness campaigns, school nutrition standards, and community mobilization interventions were implemented with moderate reach and fidelity. Challenges: Food culture, higher cost, and lower availability of healthy low-salt foods relative to unhealthy foods and salty taste preference. Coder comments: Actual salt intake not noted, just sources of exposure
Wallace, 2020 (72)	CDC	Community Coalitions for Change; engage communities in reducing obesity prevalence	4 Rural counties in Tennessee (community gardens, higher education, public schools, public and private agencies)	4 Years	Surveys, pedometers [1]; surveys, focus group interviews, ripple effect mapping (REM) [2]; REM [4]; audits [6]. Evaluation methods: physical activity survey, nutrition assessment survey, pedometer monitoring, focus group interviews to complete assessments of parks and retail food venues; audits by the evaluation team with the Physical Activity Resource Assessment; REM via the Community Capitals Framework to collect qualitative data about perceived outcomes and sustainability efforts. Other: census and health department data	Accomplishments: 67,400 Community members and 67 organizations participated; 61% reported being more physically active, 59% reported eating more fruit, and 66% reported eating more vegetables. Initiative empowered members to sustain program. Strong community engagement. Challenges: Reduced number of site assessments overtime. Coder comments: No distinction between implementation/results in different types of settings

Abbreviations: CDC, Centers for Disease Control and Prevention; DOCC, Documentation of Community Change; EFNEP, Expanded Food and Nutrition Education Program; HEAL, Healthy Eating Active Living; I+PSE, Individual Plus PSE Conceptual Framework for Action; NCI, National Cancer Institute; NHLBI, National Heart, Lung, and Blood Institute; NIH, National Institutes of Health; NSW, New South Wales, Australia; PSE, policy, systems and environment; RE-AIM, Reach, Effectiveness, Adoption, Implementation, and Maintenance; SNAP-Ed, Supplemental Nutrition Assistance Program Education; USDA, US Department of Agriculture.

a I+PSE (12) consists of 7 numbered components defined as follows: 1) strengthen individual knowledge and behavior; 2) promote community engagement and education; 3) activate intermediaries and service providers; 4) facilitate partnerships and multisector collaborations; 5) align organizational policies and practices; 6) foster physical, natural, and social settings; and 7) advance public policies and legislation. Numbers in brackets indicate which of 7 components the study addressed. Alignment with strategies describes how an aspect of the study’s initiative/intervention related to a component (eg, included an online survey, provided documentation, evaluation methods). Strategies were coded as weak if they were vaguely described, imprecise, or provided insufficient coverage of intervention activities aligned with I+PSE components.

## Results

**Figure 1:**
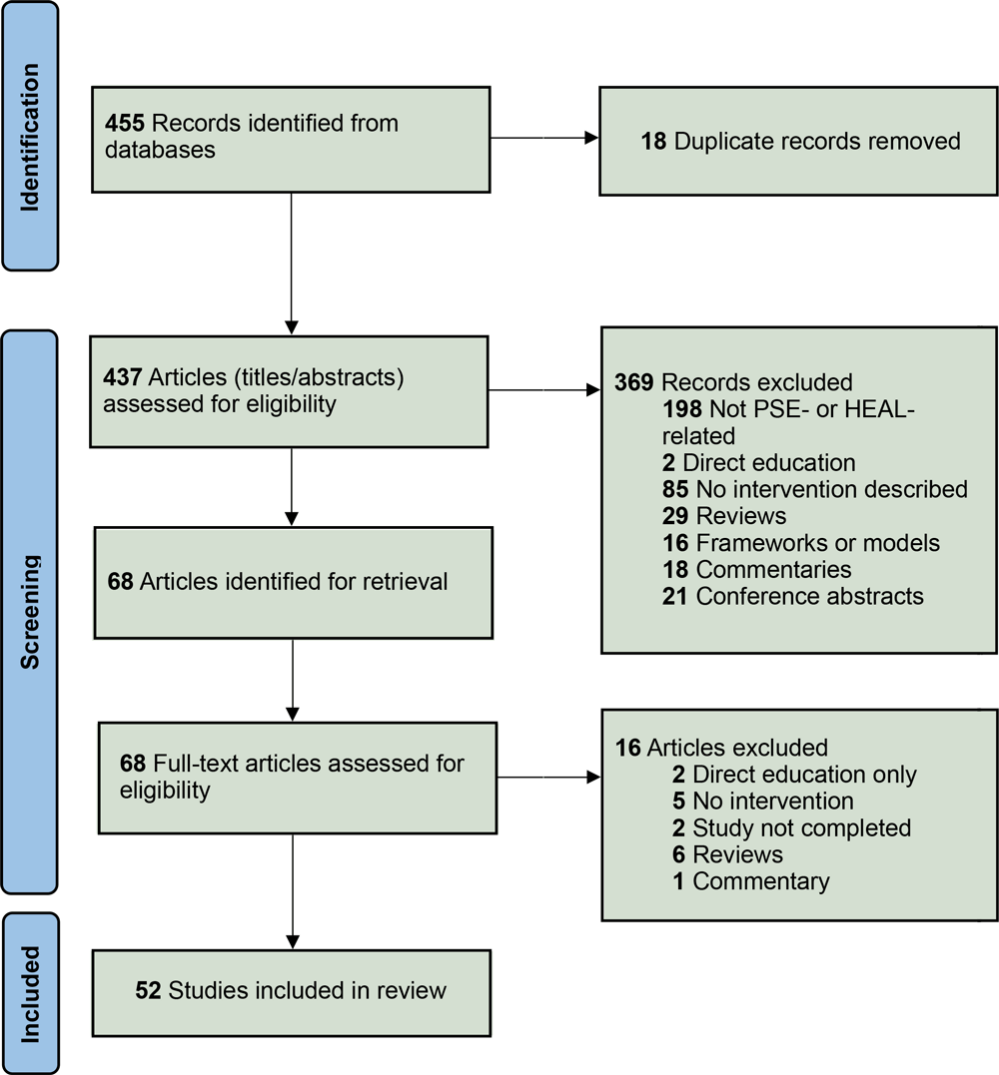
PRISMA (Preferred Reported Items for Systematic Reviews and Meta-Analyses) diagram for identification of 52 studies included in a systematic mapping review of initiatives dealing primarily with policy, systems, and environmental achievements for healthy eating and active living.

We developed a PRISMA flowchart (18) (Figure 1) noting reasons for inclusion and exclusion of articles through our 3-step review. We identified 455 articles and removed 18 duplicates. Of the remaining 437, most (n = 369) were removed because they were not PSE- or HEAL-related or because they did not describe an intervention. For articles undergoing full-text review, 16 were excluded because they described direct education, did not describe an intervention, the study was not complete, or the articles were reviews or commentaries. Of these, 52 initiatives met all inclusion criteria: 24 focused solely on healthy eating, 5 on active living, and the remaining 23 on a combination of both.

### Initiative characteristics

The most common funding sources for selected studies were federal government agencies, with CDC (n = 23), the National Institutes of Health (n = 8), and USDA (n = 7) the most prominent (Table 2). Other funding sources included foundations (n = 12) and state governments (n = 5). Some initiatives received funding from several sources. Only 2 of the 52 initiatives, both in Australia, were from researchers outside the US. Often initiatives took place in multiple settings, the most common being schools, businesses, and community organizations (Table 3). Some studied specific groups, such as people with incomes below the federal poverty level or people with high rates of obesity, and some studied racial or ethnic communities. Most initiatives (73%) were funded for 1 to 3 years.

**Table 3:** Settings (N = 52) for Policy, Systems, and Environmental Approaches for Healthy Eating and Active Living Initiatives

Setting	Number (%) of articles	Examples
Education	10 (19)	Schools, after-school programs, and early care centers
Businesses and nonprofits	9 (17)	Businesses, community-based organizations, and nonprofit organizations
Faith-based	6 (12)	Churches and related organizations
Limited food outlets	4 (8)	Convenience stores, food pantries, and farmers markets
Health care facilities	2 (4)	Hospitals and clinics
Combination of sites	21 (40)	Faith-based organizations, health care sites, schools, food stores, recreation centers, businesses and/or communities

Methods for evaluating interventions included surveys, interviews, observations, photographs, and document reviews. Seven initiatives used only surveys, 2 used only individual or group interviews, and 6 used only reviews of documents such as reports, action plans, and meeting minutes. Most (n = 34) interventions used a mixed-methods approach, and 3 reported no evaluation activities at all. Only 11 of the 52 initiatives reported using a planning or evaluation framework, 3 of which used Reach, Efficacy, Adoption, Implementation and Maintenance (RE-AIM) (19,32,46). All other frameworks mentioned were used for a single initiative (20,29,31,36,38,39,62,72).

**Figure 2:**
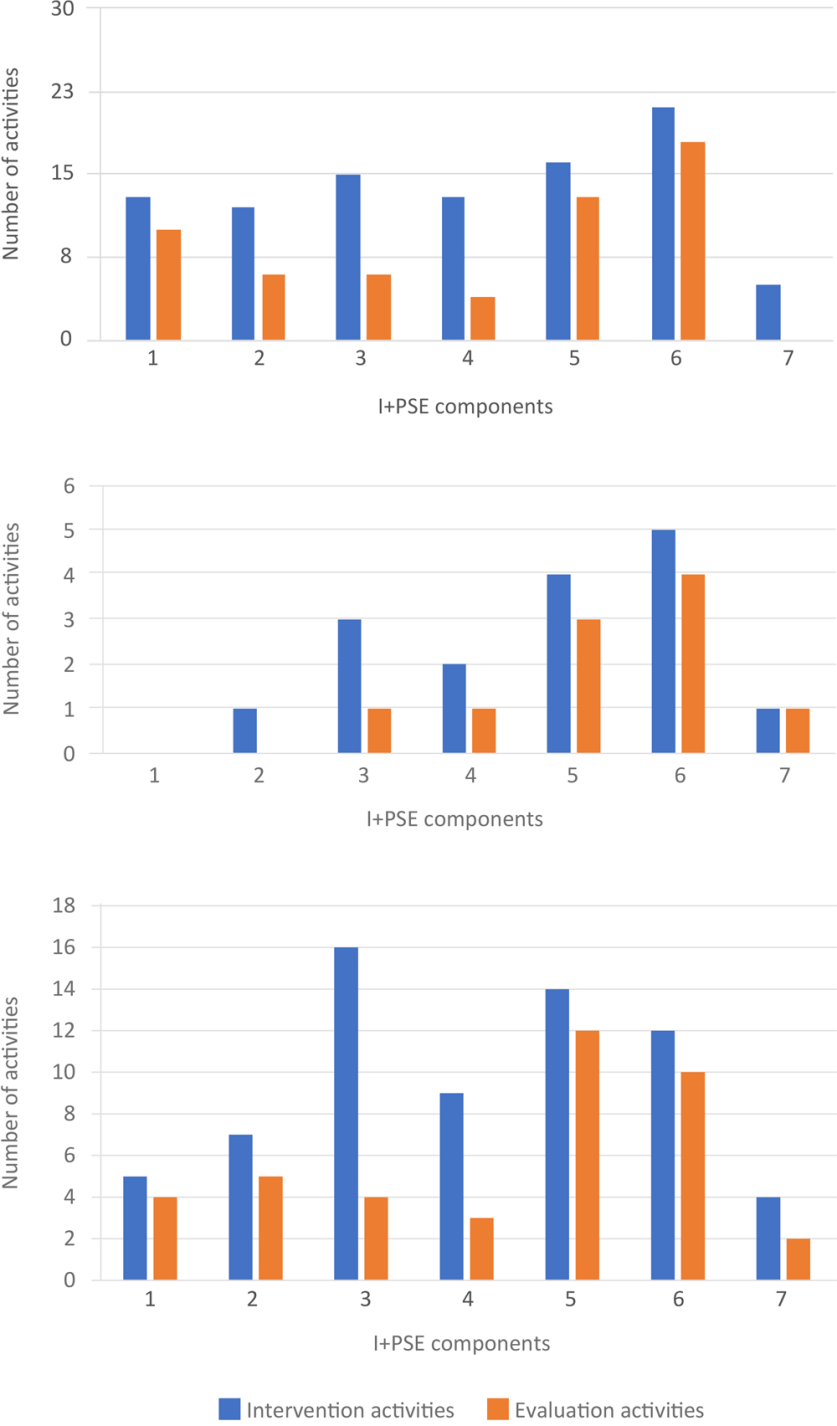
Number of activities described in 52 studies of PSE HEAL (policy, systems, and environmental healthy eating and active living) initiatives, sorted by the 7 components of the Individual Plus Policy, System, and Environmental Conceptual Framework for Action (I+PSE) (12): 1) strengthen individual knowledge and behavior, 2) promote community engagement and education, 3) educate intermediaries and service providers, 4) facilitate partnerships and multisector collaborations, 5) align organizational policies and practices, 6) sustain physical, natural and social settings, and 7) advance public policies and legislation. Graph A describes healthy eating initiatives (n = 24), B describes active living initiatives (n = 5), and C describes combined healthy eating and active living initiatives (n = 24). Initiatives may include multiple activities.

We organized HEAL interventions’ implementation and evaluation activities by the 7 I+PSE components (Figure 2). Healthy eating (n = 24) (19,22,28–30,32,34,35,38,39,43,44,46–48,52,53,56,59–62,64,66,68,71) and HEAL (n = 23) (1,20,21,24,26,27,33,36,37,40–42,45,49–51,54,57,58,63,65,67,69,72) initiatives included many intervention and evaluation activities across the 7 I+PSE components, with an emphasis on activities addressing organizational policy (I+PSE component 5) and environmental changes (I+PSE component 6) as anticipated because of the nature of this review. Those that only focused on active living (n = 5) (23,25,31,55,70) also emphasized activities addressing organizational policy (I+PSE component 5) and environmental changes (I+PSE component 6), to the near exclusion of all other components. Only 12 interventions of any kind reported attempting to advance public policies and legislative activities (I+PSE component 7) (20,26–28,31,32,49,50,54,56,59,65,71). Interestingly for PSE-focused initiatives, 20 included activities directed at the individual behavior change level (I+PSE component 1) (24,26,27,34,35,38,39,41–44,46,47,53,56,58,59,61,62,68,69,71,72). Of the 7 I+PSE components, the least likely to include evaluation activities were to “promote community engagement and education” (I+PSE component 2, 55%), “educate intermediaries and service providers” (I+PSE component 3, 32%), “facilitate partnerships and multisector collaborations” (I+PSE component 4, 33%), and “advance public policies and legislation” (I+PSE component 7, 30%).

**Table:** 

Component	Number of intervention activities	Number of evaluation activities
A. Healthy eating initiative		
1. Strengthen individual knowledge and behavior	13	10
2. Promote community engagement and education	12	6
3. Educate intermediaries and service providers	15	6
4. Facilitate partnerships and multi-sector collaborations	13	4
5. Align organizational policies and practices	16	13
6. Sustain physical, natural, and social settings	21	18
7. Advance public policies and legislation	5	0
B. Active living initiative		
1. Strengthen individual knowledge and behavior	0	0
2. Promote community engagement and education	1	0
3. Educate intermediaries and service providers	3	1
4. Facilitate partnerships and multi-sector collaborations	2	1
5. Align organizational policies and practices	4	3
6. Sustain physical, natural, and social settings	5	4
7. Advance public policies and legislation	1	1
C. Combined healthy eating and active living initiatives		
1. Strengthen individual knowledge and behavior	5	4
2. Promote community engagement and education	7	5
3. Educate intermediaries and service providers	16	4
4. Facilitate partnerships and multi-sector collaborations	9	3
5. Align organizational policies and practices	14	12
6. Sustain physical, natural, and social settings	12	10
7. Advance public policies and legislation	4	2

From the qualitative portion of the content analysis, we summarized each article’s description of accomplishments and challenges (Table 2). Initiative accomplishments were (in descending order of frequency) partnerships formed, individual behavior change, environmental and policy changes, and provision of technical assistance. Challenges were almost exclusively insufficient early engagement or investment of participating communities, resulting in resistance to initiative implementation (20,34,35,46,48,67,72). Another common challenge noted was insufficient or variable implementation because of limited resources and time and staff turnover (41,45,46,49,50,54,57,66). Lessons learned, culled from the descriptions of both accomplishments and challenges, included the importance of recruiting staff who had local trust and connections (19,33) and the value of early achievements to promote community buy-in (34,35,45,72). Authors also reported variability in the extent of implementation between different sizes of sites (36,51,57), although there was no consistent finding that larger (or smaller) sites had stronger implementation. They also reported variability on extent of implementation because of the perceived strength of collaborative partnerships, most often informally assessed through interviews or surveys (23,34–36,50,51,57,60,72).

Evaluation limitations were noted in some articles by authors and throughout our systematic coding process. As with resistance to participation in initiative activities, some authors identified reluctance of participants and stakeholders to engage in evaluation activities because of response burden and lack of buy-in (23,26–28,34,35,38,46). For many initiatives, we authors and/or other members of our review team noted limitations of evaluation tools used in terms of imprecision and insufficient coverage of intervention activities (22,33,40–42,45–52,54,55,57,58,63,65,69). Another limitation to assessing the impact of these PSE interventions was the lack of baseline or long-term follow-up measures (30,32,34,35,46,49,52,53,68). A recurring theme identified from our content analysis was the vague and limited description of evaluation tools and strategies (22,33,40–42,45–52,54,55,58,59,63,65,69).

## Discussion

Our review of 52 PSE HEAL initiatives describes their key characteristics and maps the I+PSE action components most commonly included in the intervention-to-evaluation activities. It also characterizes these initiatives’ achievements, challenges, and lessons learned. The review concludes by summarizing the evaluation matches and missed opportunities to strengthen the evidence for their outcomes.

Our review showed a gap between the most frequently reported achievement — forming partnerships — and the absence of assessments of the quality and impact of these partnerships. As the articles we reviewed stated repeatedly, weak engagement at both the coalition and community levels limited opportunities to achieve anticipated PSE Framework outcomes. Because the activities intended to foster such engagements were the least often evaluated, the influence and impact of these activities were largely unknown. Only 2 articles (49,55) mentioned measuring the quality of partnerships, but both described the use of surveys vaguely. Although the formation of coalitions and community relationships are an expected step in PSE work, the emphasis is often on documenting program implementation and outcomes. Asada and colleagues (5) concluded from their review of public health interventions that the use of valid and available evaluation tools would strengthen what we know about the impact of structural change initiatives. These include tools that measure partner engagement and collaboration. Measurement resources exist: Kegler and Swan (74) developed the Community Coalition Action Theory, which links participant engagement and resources to change community outcomes, including policy achievement. One research-tested tool to assess the effectiveness of collaborations is the Collaboration Factors Inventory offered by the Wilder Organization (75), which includes 22 success factors. Another tool is the Collaboration Framework developed by the University of Wisconsin Cooperative Extension Service, which characterizes the degree of collaboration based on a depth-of-relationship integration scale (76). I+PSE initiative leaders would do well to employ such partnership assessment tools to determine the quality and impact of their interventions.

A related finding was the lack of planning and evaluation frameworks. Brennan and colleagues (77) reviewed childhood obesity policy and environmental initiatives using the RE-AIM Framework and concluded that it was difficult to describe and summarize initiative outcomes because they lacked formal evaluations, and their multicomponent nature made it difficult to attribute outcomes to specific activities. In a scoping review of structural public health interventions, Asada and colleagues (5) reported insufficient application of theory-based evaluation frameworks and validated tools to measure change at the environmental level. Our mapping review also noted absence of planning and evaluation frameworks for all but 11 of the 52 initiatives reviewed (19,20,29,31,32,36,38,39,46,62,72). I+PSE was applied in this review because of its theoretical underpinnings, its acknowledgment and examination of the multidimensional components that support PSE change, and its adaptability to categorize HEAL initiatives. Frameworks that include assessment, engagement, and formation and strategies to strengthen coalitions and community involvement, such as I+PSE, will support the effectiveness of PSE initiatives.

We also found the limited funding period of just 1 to 3 years required by government and foundation funding sources to be disappointing but not unexpected. The time needed to establish or strengthen existing coalitions, assess needs, and prioritize PSE strategies can be lengthy but is essential for success (45,78). For example, after examining efforts to improve maternal and child health outcomes in 14 North Carolina counties, Schaffer and colleagues (8) concluded that more upfront time was necessary to form community action teams able to sustain community engagement. After Holston and colleagues (45) implemented multilevel obesity prevention interventions in 3 rural Louisiana parishes, they recommended identifying attainable early successes, not only to engage and strengthen partnerships but also in recognition of the time it takes for significant PSE change to be realized. When funding periods cannot be lengthened, funders, researchers and practitioners must identify realistic outcomes for these brief timeframes, such as the development of strong community linkages.

The need for technical assistance for I+PSE implementation and evaluation has been widely reported (2), including by those using the SNAP-Ed Evaluation Framework (79–81). Naja-Riese and colleagues (9) noted in their review of national SNAP-Ed results that implementing agencies still focused most of their activities and evaluation measures at the traditional individual-change level, despite the intended focus on PSE change. They posited that practitioners need technical assistance to learn to implement and measure multisector activities. Our review found similarly that delivery of individual behavior change activities was the second most frequent accomplishment. Herman and colleagues (82) noted from interviews with state and regional public health nutrition teams working in maternal and child health the need for technical assistance and the value it garnered in the development of PSE action plans. In our review, 7 initiatives provided technical assistance to coalition members leading PSE efforts or intermediary service providers (28,45,46,48,54,64,70), but none described in any detail recipient response to the value or effectiveness of the technical assistance. Assessing and addressing I+PSE implementation and evaluation capacity and readiness for teams leading the initiative is critical for success but was not even described in any of the 52 articles we reviewed.

Using a mapping review approach allowed us to visualize the match between intervention and evaluation activities across the 7 distinct components of I+PSE. Ours is the first attempt, to our knowledge, to examine and characterize PSE HEAL initiatives with the detail this I+PSE provides and with an evaluation focus. We found the framework to be adaptable and applicable to a variety of HEAL initiatives, and unlike other frameworks, it acknowledges and includes assessment of individual behavior change resulting from PSE approaches, which are common activities in PSE initiatives.

However, our review was not exhaustive. We did not seek out articles related to those in our review that did not meet the inclusion criteria themselves, nor did we search the gray literature. We also did not report funding amounts, which could have influenced the scope and reach of these initiatives, including their evaluation activities and results. Funding amounts would be an important component in future reviews, as would a more in-depth examination of the relationships between strength of coalition and community engagement and achievement of PSE outcomes. Future reviews also could explore the perceived value and application of evidence-based practice and practice-based evidence (83) in PSE HEAL initiatives for which there is no “best” intervention and evaluation design because of diversity in aims and approaches, and multicomponent complexity (5). Future research also could examine funding sources, I+PSE activities and evaluation components, and outcomes for different audiences (eg, by school level, initiative setting) to identify patterns specific to those audiences. Finally, despite the breadth of our search terms, only 2 initiatives were identified outside the US (48,83), and both of these were located in Australia. This limits the generalizability of our results.

As poor dietary patterns, sedentary lifestyles, diet-related chronic diseases, and associated health care costs increase in the US (84), the need is urgent for greater focus on and investment in learning how individual PSE change approaches can be optimized to advance healthy eating and active living across households, communities, and populations. Our mapping review shows potential gaps and suggests opportunities to advance research and practice in formulating, implementing, and evaluating PSE HEAL initiatives. Future initiatives should give special attention to closing the gap between activating community and service provider partnerships and evaluating the quality and impact of these relationships, because outcomes will rely on the strength of these relationships.

## Post-Test Information

To obtain credit, you should first read the journal article. After reading the article, you should be able to answer the following, related, multiple-choice questions. To complete the questions (with a minimum 75% passing score) and earn continuing medical education (CME) credit, please go to http://www.medscape.org/journal/pcd. Credit cannot be obtained for tests completed on paper, although you may use the worksheet below to keep a record of your answers.

You must be a registered user on http://www.medscape.org. If you are not registered on http://www.medscape.org, please click on the “Register” link on the right hand side of the website.

Only one answer is correct for each question. Once you successfully answer all post-test questions, you will be able to view and/or print your certificate. For questions regarding this activity, contact the accredited provider, CME@medscape.net. For technical assistance, contact CME@medscape.net. American Medical Association’s Physician’s Recognition Award (AMA PRA) credits are accepted in the US as evidence of participation in CME activities. For further information on this award, please go to https://www.ama-assn.org. The AMA has determined that physicians not licensed in the US who participate in this CME activity are eligible for AMA PRA Category 1 Credits™. Through agreements that the AMA has made with agencies in some countries, AMA PRA credit may be acceptable as evidence of participation in CME activities. If you are not licensed in the US, please complete the questions online, print the AMA PRA CME credit certificate, and present it to your national medical association for review.

## Post-Test Questions

### Study Title: Partnerships and Community Engagement Key to Policy, Systems, and Environmental Achievements for Healthy Eating and Active Living: a Systematic Mapping Review

#### CME Questions

1. Which one of the following statements regarding characteristics of the collective research reviewed in the current study of the Policy, Systems, and Environmental Healthy Eating and Active Living (PSE HEAL) initiatives is most accurate?
  - Most studies were funded by the federal government
  - Half the studies were conducted in Europe
  - Just 8% of the interventions were conducted in multiple settings
  - The average duration of the intervention period was 5 years
2. Which one of the following Individual plus PSE (I+PSE) action components was most commonly followed in PSE HEAL healthy eating and active living initiatives in the current study?
  - Strengthen individual knowledge and skills
  - Promote community education and engagements
  - Foster physical, natural, and social settings
  - Advance public policy and legislation
3. What was observed as the greatest accomplishment cited in PSE HEAL research?
  - Development of new partnerships
  - Reduced rates of disease such as hypertension and diabetes
  - Early engagement with community partners
  - Development of new evaluation tools
4. Which one of the following was one of the most common lessons learned from the collective PSE HEAL research?
  - Emphasize engagement with policy leaders
  - Develop evaluation frameworks to drive the intervention methodology
  - Maintain flexibility in implementation across different sites
  - Early recruitment of staff with local trust and connections
